# Joint predictors of antenatal care contacts and timing of antenatal care initiation among women of reproductive age in Ethiopia

**DOI:** 10.1371/journal.pone.0330873

**Published:** 2025-11-07

**Authors:** Abebew Aklog Asmare, Awoke Seyoum Tegegne, Denekew Bitew Belay

**Affiliations:** 1 Department of Statistics, College of Science, Bahir Dar University, Bahir Dar, Ethiopia; 2 Department of Statistics, College of Natural and Computational Science, Mekdela Amba University, Tuluawlyia, Ethiopia; 3 School of Health Systems and Public Health, Faculty of Health Science, University of Pretoria, Pretoria, South Africa; Kandahar University, Faculty of Medicine, AFGHANISTAN

## Abstract

**Background:**

Maternal and neonatal mortality remain major public health challenges, particularly in low-income countries like Ethiopia. Antenatal care (ANC) plays a vital role in improving maternal and neonatal outcomes. This study aimed to jointly assess the predictors of *ANC contacts* (defined as a woman receiving four or more contacts during pregnancy) and *timing of ANC initiation* (defined as a woman starting ANC early (during the first trimester)) using a bivariate binary logistic regression model.

**Methods:**

Data were drawn from the 2019 Ethiopian Mini Demographic and Health Survey (EMDHS), conducted by the measures of the DHS Program. A total of 5,492 weighted women aged 15–49 years with recent births were included. A bivariate binary logistic regression model was employed to simultaneously examine the two ANC outcomes, taking into account their potential interdependence. Adjusted odds ratios (AORs) with 95% confidence intervals (CIs) were reported.

**Results:**

The prevalence of completing four or more ANC visits and timely ANC initiation was 59.7% and 19.8%, respectively. Among 5,492 ever-married women, timely ANC initiation and completing four or more ANC visits were significantly associated. Maternal age, region, maternal education, wealth index, and number of children ever born were identified as **joint predictors** affecting both outcomes. For example, compared to women from poor households, those from middle-income households had higher odds of receiving four or more ANC contacts (AOR = 1.391; 95% CI: 1.121–1.726) and initiating ANC early (AOR = 2.047; 95% CI: 1.669–2.511).

**Conclusion:**

The findings highlight significant socioeconomic and geographic disparities in ANC utilization in Ethiopia. Integrating both outcomes into a joint modeling framework provides a more comprehensive understanding of maternal health service use. Targeted interventions are needed to promote early and adequate ANC, particularly among poor and rural populations.

## Introduction

Maternal and neonatal mortality are major public health issues that reflect a country’s overall healthcare system and socioeconomic progress [1,2]. Approximately 700 maternal deaths per day will occur in 2023, most of which are preventable [3]. An estimated 260,000 women will have died during or shortly after pregnancy and childbirth by 2023 [3]. Maternal mortality is shockingly high [4]. The vast majority of these deaths (almost 90%) occurred in low- and middle-income countries and might have been avoided [3,4]. Sub-Saharan Africa and Southern Asia jointly accounted for around 87% of global maternal mortality in 2023. Sub-Saharan Africa alone accounted for about 70% of maternal mortality, with Southern Asia accounting for approximately 17% [3]. As a result, ANC continues to be an important strategy for lowering maternal mortality, and it can be provided in lower-level health facilities [5,6]. Early ANC contacts can significantly minimize maternal mortality by detecting problems early and allowing for HIV screening, syphilis testing, and treatment [7].

The third Sustainable Development Goal (SDG) focuses on reducing maternal mortality and enhancing women’s healthcare, with the goal of lowering the global maternal mortality rate (MMR) to 70 per 100,000 live births by 2030 [3,7,8]. Between 2000 and 2023, the global MMR decreased by around 40% [3]. Despite a decrease in MMR from 412 in 2000–195 per 100,000 live births in 2023 in Ethiopia, the risk of maternal-related death remains high, with one in every 64 women at risk, compared to the SDG objective [3]. This suggests that additional efforts are required to accomplish the SDGs during the coming decade. Regular ANC from a trained clinician lowers maternal mortality by about 20% [9]. According to the 2019 EMDHS, 74% of women who gave birth in the five years before the survey had ANC from a skilled provider, with 85% in urban areas and 70% in rural areas [10]. Furthermore, the EMDHS 2019 showed that during their ANC contacts, 88% of pregnant women had their blood pressure checked, 79% had blood samples taken, 74% had urine samples obtained, and 61% got dietary counseling [11].

The use of health facilities is directly related to ANC contacts, with adequate ANC including both the use of services and the quality of care delivered [12]. According to the 2019 EMDHS, only 28% of women received their first ANC visit in the first trimester, 32% in the fourth or fifth month, and 12% in the sixth or seventh month of pregnancy. Only 2% of women began obtaining ANC in the eighth month or later [11].

Attending at least four ANC sessions during pregnancy is critical since it has been demonstrated to lower maternal mortality and morbidity while promoting safe parenthood and increasing maternal health outcomes [13,14]. The World Health Organization (WHO) recommends that the first ANC visit take place before 12 weeks of pregnancy, coupled with specified visit content (clinical assessment, blood pressure measurement, urine and blood tests, HIV and syphilis testing, iron and folic acid supplementation, tetanus toxoid vaccination, nutritional counseling, and health education on pregnancy danger signs and birth preparedness), to improve women’s care experiences and minimize perinatal death [15]. However, Ethiopia has failed to achieve its aim of four or more ANC contacts [16]. Despite advances in the frequency of contacts and the quality of services, many Ethiopian women continue to postpone their initial ANC appointment, missing out on vital care.

Several studies conducted in Ethiopia over the last year have specifically examined the characteristics associated with ANC contacts and the completion of four or more contacts during pregnancy [17–19], while others have focused on the timing of ANC initiation [10,20–23]. Although ANC coverage is high, the quality and content of care may still be inadequate. While many studies have looked into the factors that influence ANC contacts in Ethiopia, with a particular emphasis on the number of ANC contacts or the timing of ANC initiation, these outcomes have typically been analyzed separately using separate univariate logistic regression models. Such techniques fail to account for the potential interaction of these two important outcomes of ANC use. To our knowledge, no prior work has concurrently modeled ANC contacts (4 or more contacts) and timely ANC commencement (during the first trimester) using nationally representative Ethiopian data. This article fills that gap by using a bivariate binary logistic regression model that assesses both outcomes at the same time while accounting for any potential association. The bivariate binary logistic regression model contributed significantly to this study by capturing the correlation between the two ANC outcomes, timely initiation and ANC contacts, which are likely influenced by similar factors. By analyzing them together, the model provided more precise estimates and a deeper understanding of shared and unique predictors, revealing patterns that might be missed with separate univariate models. This joint modeling approach allowed us to better identify policy-relevant determinants and support more integrated maternal health interventions. Therefore, the objective of this study was to analyze the joint influence of maternal, household, and community-level factors on both ANC contacts and timing of ANC initiation among Ethiopian women aged 15–49 years who had a live birth within five years before the 2019 EMDHS, using a bivariate binary logistic regression model to account for the correlation between the two ANC components.

## Materials and methods

### Data source, study design, and setting

This study utilizes data from the most recent EMDHS conducted in 2019. The data was accessed from the DHS website (https://www.dhsprogram.com/data/dataset_admin/login_main.cfm) after submitting a reasonable request. The 2019 EMDHS is a cross-sectional survey carried out in Ethiopia, which is located in the Horn of Africa. Ethiopia covers a total area of 1,100,000 square kilometers and lies between latitudes 3° and 15° north and longitudes 33° and 48° east.

### Population and sampling procedure

The study included all pregnant women who were pregnant within five years prior to the survey. The 2019 EMDHS used a two-stage stratified cluster sampling method, with sample households selected from cluster enumeration areas (EAs). In the first stage, 305 EAs (93 urban and 212 rural) were selected in proportion to EA size, with independent selection at each sampling level. In the second stage, 30 households were systematically chosen from each cluster, including the option to select newly formed households from updated lists. Additional details on the data collection, sampling, and questionnaires used in the survey can be found in the 2019 EMDHS Report [24].

### Inclusion and exclusion criteria

**Fig 1:**
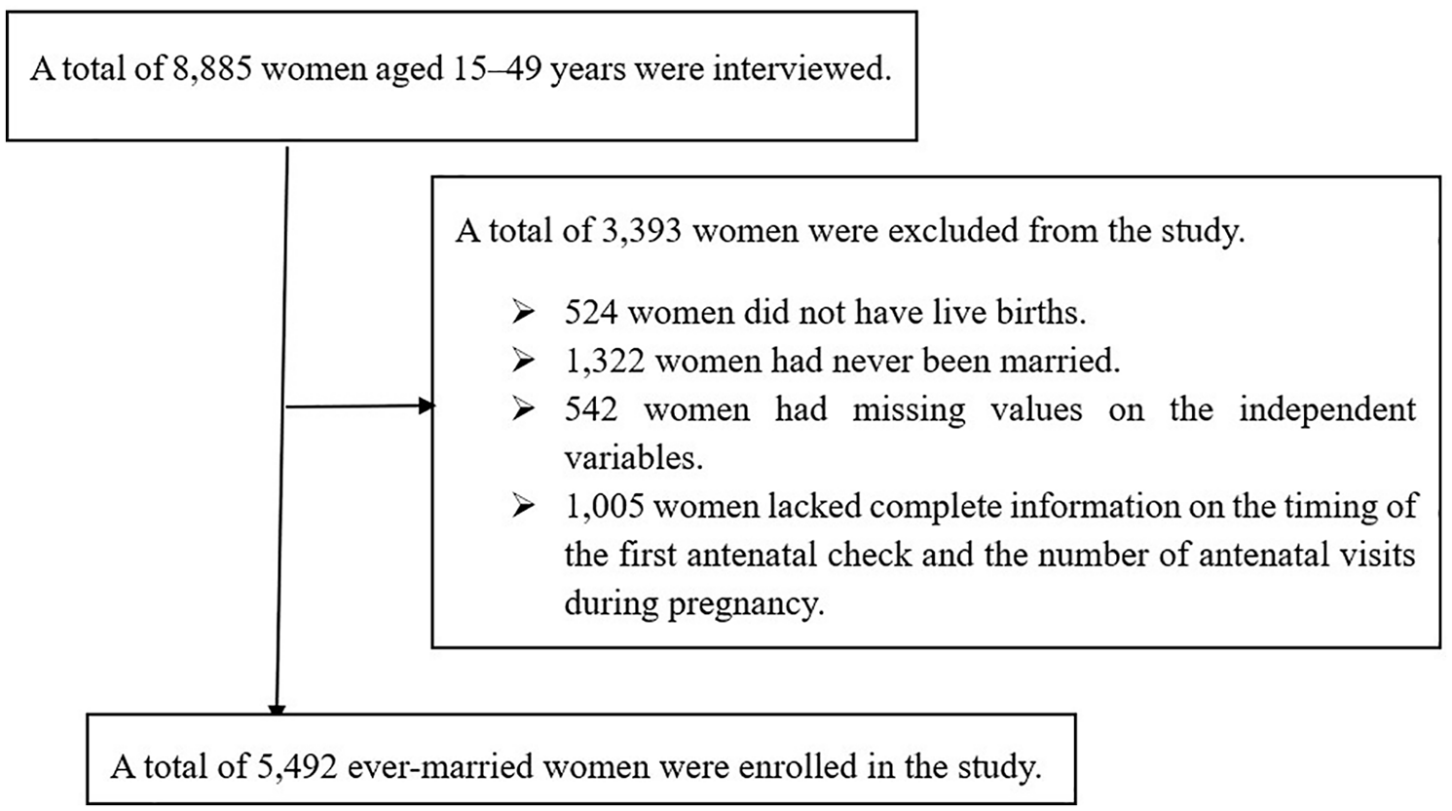
Sample selection procedures. depicts the sampling and data filtration process for the 2019 EMDHS. It follows the progression from the national sample to the final analytic cohort, emphasizing exclusion criteria and the reason for selecting a viable study group centered on ANC usage.

Women aged 15–49 years who had a live birth in the five years preceding the 2019 EMDHS and who had complete data on the number and timing of ANC contacts. Women who had missing or inconsistent information on ANC visit frequency or timing, or other key variables required for the analysis, were excluded (see **Fig 1**).

### Study variables and measurements

#### Outcome variables.

In this study, two binary outcome variables were considered: antenatal care (ANC) contacts [25,26] and timing of ANC initiation (TANI) [27].


$$ANC=\left\{ \begin{array}{c} \;\;Yes=\text{1},IfawomenattendedfourormoreANCcontactsorvisits\\ \;\;No=\text{0},otherwise \end{array}\right.$$

$$\text{Timing of ANC initiation}=\left\{ \begin{array}{c} \;\;Yes=\text{1},IfawomanstartedANCearly(duringthefirsttrimester)\\ \;\;No=\text{0},otherwise \end{array}\right.$$

### Independent variables

**Fig 2:**
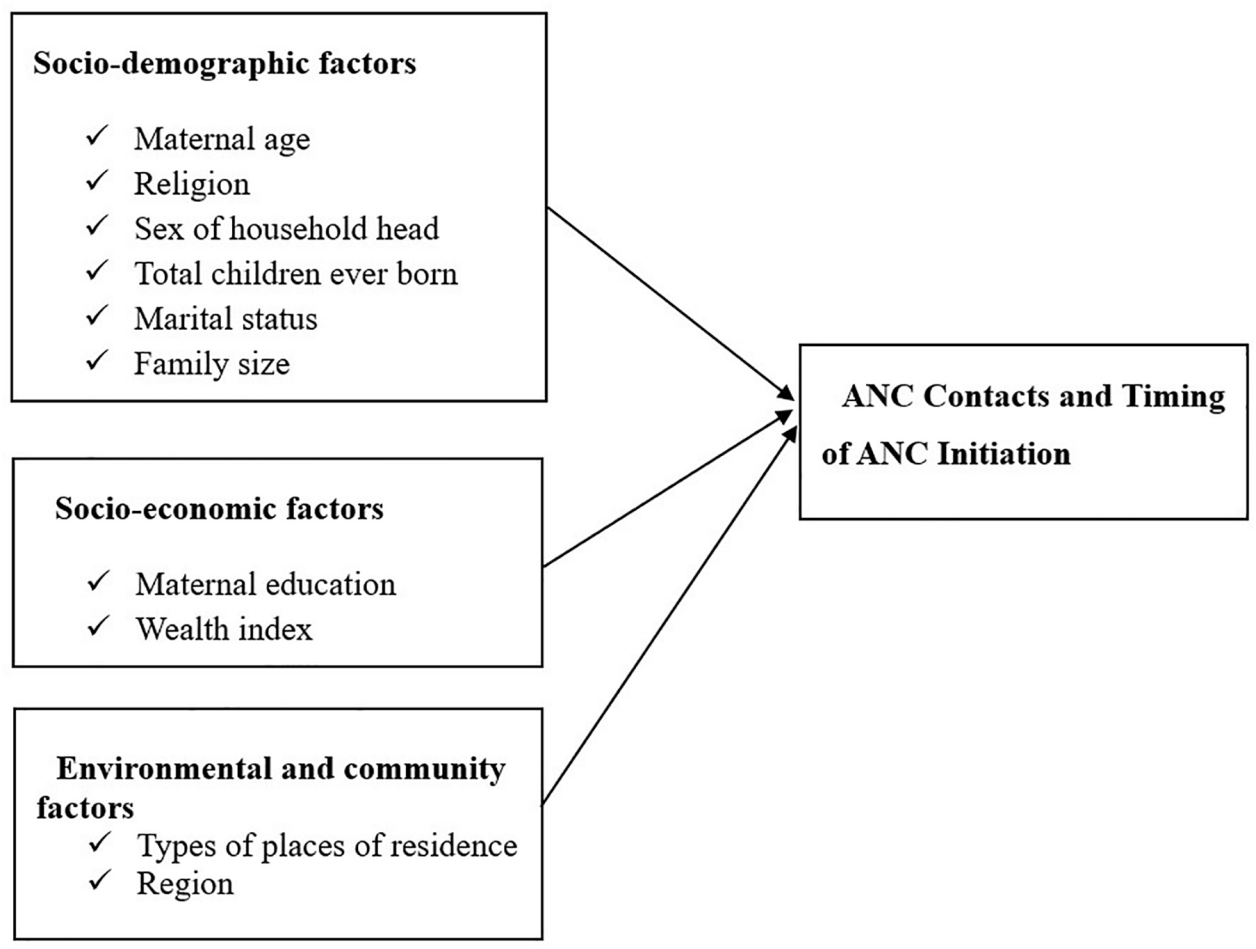
Conceptual diagram of predictors associated with outcome variables. Shows the basic structure that supports this study, illustrating the expected associations between factors related to mothers, households, and their communities, and the two outcomes of ANC: early initiation and having the optimal number of ANC contacts. This structure guided variable selection and the multivariate modeling method.

The study considered a range of independent variables that have been identified in prior literature as influencing maternal healthcare utilization (see **Fig 2**). These included maternal age group, categorized into seven categories (15–19, 20–24, 25–29, 30–34, 35–39, 40–44, and 45–49 years), and region, covering all administrative regions of Ethiopia to capture geographic diversity. The place of residence was classified as either urban or rural. The mother’s education level was grouped into four categories: no education, primary, secondary, and higher education. Religion was also included as a categorical variable comprising Orthodox, Catholic, Muslim, Protestant, and others.

Household-related factors included family size, grouped into less than 4, 4–8, and more than 8 members, and the sex of the household head (male or female). Socioeconomic status was captured using the wealth index, which is a standard DHS-derived indicator divided into five quintiles, but in this article, it can be poor, middle, or rich. Reproductive history was represented by the total number of children ever born, categorized into 1, 2–3, and more than 3. Marital status was classified as single, married, widowed, or divorced. These variables were selected based on theoretical relevance and prior empirical evidence from global studies on ANC utilization [11,28–32].

### Statistical analysis

#### Logistic regression model.

Bivariate binary logistic regression is a statistical model used to estimate the effect of predictors on two binary outcome variables. In this study, let $y_{1i}andy_{2i}$ represent the dichotomous outcomes of ANC contacts and the timing of ANC initiation for the $i^{th}$ woman of reproductive age, respectively. For the dichotomous outcomes $y_{ji}$ and a vector of independent variables x, the bivariate binary logistic regression model is expressed as follows [33]:


$$\pi_{j(X)}=\frac{e^{\beta_{j\text{0}}+\beta_{j\text{1}}X_{\text{1}}+\beta_{j\text{2}}X_{\text{2}}+\ldots +\beta_{jp}X_{p}}}{{\text{1}+e}^{\beta_{j\text{0}}+\beta_{j\text{1}}X_{\text{1}}+\beta_{j\text{2}}X_{\text{2}}+\ldots +\beta_{jp}X_{p}}}=\frac{e^{X\beta_{j}}}{\text{1}+e^{X\beta_{j}}},j=\text{1},\text{2}$$ (1)

Where $\pi_{j(X)}=P(y_{ji}=\frac{\text{1}}{x})$, the probability of the $i^{th}$ reproductive-aged women using ANC contacts ($y_{1i}$), and using the timing of ANC initiation ($y_{2i}$) given other predictors x. Consistently, the logit (log odds) that marked a linear association with independent variables can be stated as:


$$logit\left[P\left(Y_{ji}=\frac{\text{1}}{X}\right)\right]=\beta_{j\text{0}}+\beta_{j\text{1}}X_{\text{1}}+\beta_{j\text{2}}X_{\text{2}}+\ldots +\beta_{jp}X_{p}={X\beta }_{j},j=\text{1},\text{2}$$ (2)

Odds ratio is the best method that is used to measure the relationship between categorical variables in the logistic regression model. It is the proportion of odds defined as:


$${OR}_{j}=\frac{\frac{\pi_{j}(X_{\text{1}})}{\text{1}-\pi_{j}(X_{\text{1}})}}{\frac{\pi_{j}(X_{\text{2}})}{\text{1}-\pi_{j}(X_{\text{2}})}}$$ (3)

A bivariate binary logistic regression model is used to analyze two binary outcome variables simultaneously, allowing the assessment of their relationship with other explanatory variables [34,35]. This model is suitable for modeling marginal likelihoods as a function of predictor variables, while also evaluating the relationship between ANC contacts and the timing of ANC initiation among women of reproductive age.

We first examined the association between timely ANC initiation and number of ANC contacts using unadjusted odds ratios (COR). Subsequently, multivariable logistic regression was used to identify predictors affecting both outcomes simultaneously (joint predictors) and those affecting each outcome independently, accounting for the correlation between the two dependent variables.

This model is appropriate because it accounts for the potential correlation between the two ANC outcomes and improves estimation efficiency compared to separate univariate models. A simplified explanation of the model and its rationale is described in the cited literature [35,36] and referenced online resources (https://www.researchgate.net/publication/342676296_Bivariate_Response_Logistic_Regression_for_Categorical_Data. Initially, SPSS Version 27 was used for data importation and management, while the final analysis was conducted using the **VGAM** (Vector Generalized Additive Models) package in R version 4.0.5. This package enables simultaneous modeling of multiple correlated binary responses. [37]. VGAM offers functions for fitting vector, generalized linear, and additive models.

### The goodness of the test

Before fitting the model, its adequacy or goodness of fit must be evaluated. This can be done using the classification rate, specifically the “correct classification rate,” which is the proportion of correct predictions out of the total observations. A high correct classification rate indicates that the fitted model accurately estimates the data [38].

### Ethics approval and consent to participate

This study was built on the analysis of openly accessible secondary data, with all identifier information removed. The Institutional Review Board (IRB) of ICF Macro at Fairfax, Virginia, in the USA, reviewed and approved the MEASURE DHS Project Phase Three. The 2010–2018 DHSs are considered under that approval. The IRB of ICF Macro complied with the United States Department of Health and Human Services requirements for the “Protection of Human Subjects” (45 CFR 46). Most importantly, the informed consent statement emphasizes that participation is voluntary; that the respondent may refuse to answer any question, decline any biomarker test, or terminate participation at any time; and that the respondent’s identity and information will be kept strictly confidential. In addition, written informed consent was obtained from a parent or guardian for participants under 16 years old. ICF Macro permitted the authors to use the data. The full details of the ethical approvals can be found at http://dhsprogram.com, and the data can be found on https://www.dhsprogram.com/data/dataset_admin/login_main.cfm. We confirm that all methods were carried out according to the relevant guidelines and regulations.

## Results

### Exploratory analysis

#### Characteristics of independent variables.

The results in Table 1 indicate that approximately 80% of reproductive-age women were from three regions in Ethiopia: Amhara (1,033; 18.8%), SNNPR (1,104; 20.1%), and Oromia (2,204; 40.1%). Most of the study participants resided in rural areas (4,137; 75.3%). More than half of the women were uneducated (2,942; 53.6%). Additionally, 45.6% of households had a poor wealth index, and 86.4% were headed by a male. Most households (3,131; 57.0%) had four to eight family members, and more than half of the women (2,810; 51.2%) had given birth to more than three children. The majority of reproductive-age women (5,315; 93.0%) were married.

**Table 1:** Frequency and percentage distribution of the independent variables.

Predictors	Charterparties	Weighted frequency (%)
Maternal age	15–19	263 (4.8)
	20–24	1021 (18.6)
	25–29	1749 (31.8)
	30–34	1186 (21.6)
	35–39	798 (14.5)
	40–44	366 (6.7)
	45–49	109 (2.0)
Region	Tigray	369 (6.7)
	Afar	85 (1.6)
	Amhara	1033 (18.8)
	Oromia	2204 (40.1)
	Somali	405 (7.4)
	Benishangul Gumz	67 (1.2)
	SNNPR	1104 (20.1)
	Gambela	25 (0.4)
	Harari	16 (0.3)
	Addis Ababa	154 (2.8)
	Dire Dawa	29 (0.5)
Type of place of residence	Urban	1355 (24.7)
	Rural	4137 (75.3)
Maternal education	No education	2942 (53.6)
	Primary	1948 (35.3)
	Secondary	408 (7.4)
	Higher	194 (3.5)
Religion	Orthodox	2905 (38.2)
	Catholic	17 (0.3)
	Protestant	1452 (26.4)
	Muslim	1838 (33.5)
	Other	89 (1.6)
Family size	Less than 4	1623 (29.5)
	4–8	3131 (57.0)
	8 and more	738 (13.4)
Sex of household head	Male	4744 (86.4)
	Female	748 (13.6)
Wealth index	Poor	2504 (45.6)
	Middle	1040 (18.9)
	Rich	1948 (35.3)
Total number of children ever born	Only one	820 (14.9)
	2–3	1862 (33.9)
	More than 3	2810 (51.2)
Martial status	Single	126 (2.3)
	Married	5155 (93.9)
	Widowed	66 (1.2)
**Predictors**	**Charterparties**	**Weighted frequency (%)**
Martial status	Divorced	146 (2.7)

Key: SNNPR represents the southern nation nationality and people region

### Characteristics of dependent variables

Out of a weighted sample of 5,492 reproductive-age women included in this study, 3,277 (59.7%) had four or more ANC contacts, and 1,088 (19.8%) began their ANC contacts in the first trimester (see Table 2).

**Table 2:** Frequency and percentage distribution of the outcome variables.

Outcome variables	Categories	Frequency (%)
Optimal ANC contacts	Less than 4 contacts	2215 (40.3)
	4 or more contacts	3277 (59.7)
Timing of ANC initiation	After first trimester	4404 (80.2)
	First trimester	1088 (19.8)

Table 3 presents the joint and marginal probabilities of ANC contacts and the timing of ANC initiation for reproductive-age women, along with the odds ratio (OR). The odds ratio is a standard measure used to assess the relationship between the two dichotomous outcomes. An OR of one indicates statistical independence between the outcomes [33]. In this case, the OR was 2.564, suggesting a deviation from unity and indicating a potential dependency between the two outcomes [33]. Consequently, it is crucial to fit a bivariate binary logistic regression model to analyze the joint probability of ANC contacts and the timing of ANC initiation while accounting for the possible interdependence between these outcomes. Table 6 provides the results of the bivariate logistic regression analysis, which examines ANC contacts and the timing of ANC initiation in reproductive-age women to other predictors.

**Table 3:** Joint and marginal probability of ANC contacts and timing of ANC initiation.

		ANC contacts		Marginal of timing of ANC initiation	Odds ratio (OR)
		Less than 4 contacts	4 or more contacts		
**Timing of ANC initiation**	**After the first trimester**	1957 (0.356)	2447 (0.446)	4404 (0.802)	2.564
	**First trimester**	258 (0.047)	830 (0.151)	1088 (0.198)	
**Marginal of ANC contacts**		2215 (0.403)	3277 (0.597)	5492 (1.000)	

Key: Numbers in each cell are the frequency, and their probability is in parentheses.

**Table 4:** Model Comparison Summary.

Models	Model comparison criteria			
	**Deviance**	**LLR**	**AIC**	**BIC**
Null model	12709.37	−6354.69	12715.37	12735.33
Full model	11629.15	−5814.57	11771.15	12243.32

**Table 5:** Parameter estimation of bivariate binary logistic regression modeling of ANC contacts and timing of ANC initiation among women.

Predictors	Crude Odds Ratio (COR)				Adjusted Odds Ratio (AOR)			
	_ANC contacts_		Timing of ANC initiation		_ANC contacts_		Timing of ANC initiation	
	COR (95% CI)	P -value	COR (95% CI)	P- value	AOR (95% CI)	P -value	AOR (95% CI)	P -value
**Intercept**	–	–	–	–	0.16 (0.08–0.32)	0.000	0.42 (0.21–0.82)	0.011
**Maternal age**								
15–19 (Ref.)	Ref.		Ref.		Ref.		Ref.	
20–24	2.18 (1.66–2.88)	0.000	1.44 (1.02–2.03)	0.038	1.38 (0.95–2.00)	0.091	1.38 (1.02–1.87)	0.036
25–29	2.70 (2.07–3.52)	0.000	1.13 (0.81 - 1.58)	0.463	1.72 (1.17–2.52)	0.006	1.56 (1.15–2.13)	0.005
30–34	2.66 (2.02–3.50)	0.000	1.12 (0.80–1.58)	0.504	2.25 (1.48–3.42)	0.000	1.45 (1.04–2.03)	0.030
35–39	1.89 (1.43–2.51)	0.000	0.95 (0.67–1.37)	0.800	2.25 (1.44–3.51)	0.000	1.02 (0.71–1.45)	0.931
40–44	1.84 (1.33–2.54)	0.000	0.58 (0.37–0.91)	0.017	1.52 (0.90–2.58)	0.120	0.97 (0.66–1.43)	0.873
45–49	1.26 (0.80–1.97)	0.384	0.65 (0.34–1.24)	0.191	1.57 (0.75–3.28)	0.228	0.70 (0.42–1.16)	0.166
**Region**								
Tigray	0.43 (0.26–0.72)	0.001	0.45 (0.30–0.65)	0.000	0.92 (0.60–1.40)	0.697	0.66 (0.38–1.15)	0.144
Afar	0.24 (0.13–0.45)	0.000	0.20 (0.10–0.38)	0.000	1.06 (0.52–2.15)	0.874	0.51 (0.26–1.01)	0.053
Amhara	0.25 (0.16–0.41)	0.000	0.39 (0.28–0.55)	0.000	1.05 (0.71–1.54)	0.822	0.49 (0.29–0.82)	0.006
Oromia	0.24 (0.15–0.38)	0.000	0.19 (0.13–0.26)	0.000	0.69 (0.47–1.02)	0.061	0.47 (0.28–0.78)	0.004
Somali	0.18 (0.11–0.30)	0.000	0.05 (0.03–0.09)	0.000	0.34 (0.19–0.62)	0.000	0.39 (0.22–0.68)	0.000
Benishangul Gumz	0.38 (0.19–0.75)	0.006	0.26 (0.13–0.50)	0.000	0.82 (0.40–1.68)	0.588	0.62 (0.30–1.31)	0.210
SNNPR	0.19 (0.12–0.30)	0.000	0.16 (0.12–0.23)	0.000	0.50 (0.33–0.75)	0.000	0.34 (0.20–0.60)	0.000
Gambela	0.15 (0.06–0.37)	0.000	0.30 (0.11–0.80)	0.016	0.76 (0.27–2.15)	0.601	0.20 (0.07–0.52)	0.001
Harari	0.24 (0.08–0.70)	0.000	0.53 (0.18–1.54)	0.246	1.31 (0.41–4.14)	0.647	0.30 (0.10–0.97)	0.044
Addis Abeba	Ref.		Ref.		Ref.		Ref.	
Dire Dawa	0.46 (0.18–1.16)	0.100	0.66 (0.30–1.48)	0.314	1.59 (0.66–3.87)	0.303	0.63 (0.23–1.71)	0.366
**Maternal education**								
No education	Ref.		Ref.		Ref.		Ref.	
Primary	1.36 (1.21–1.53)	0.000	1.89 (1.63–2.19)	0.000	1.13 (0.94–1.36)	0.186	1.38 (1.19–1.59)	0.000
Secondary	1.06 (0.85–1.65)	0.327	2.88 (2.28–3.64)	0.000	0.90 (0.68–1.20)	0.481	2.45 (1.84–3.25)	0.000
Higher	2.26 (1.90–5.27)	0.000	3.42 (2.49–6.93)	0.000	1.60 (1.12–2.29)	0.010	2.94 (1.91–4.52)	0.000
**Place of residence**								
Urban	Ref.		Ref.		Ref.		Ref.	
Rural	0.37 (0.33–0.44)	0.000	0.58 (0.51–0.66)	0.000	0.79 (0.65–0.96)	0.016	0.88 (0.74–1.04)	0.124
**Religion**								
Orthodox	Ref.		Ref.		Ref.		Ref.	
Catholic	0.21 (0.07–0.60)	0.000	0.23 (0.14–2.31)	0.981	0.11 (0.00–8.26)	0.987	0.35 (0.12–1.06)	0.062
Protestant	0.91 (0.71–1.81)	0.401	0.82 (0.74–1.62)	0.198	0.93 (0.72–1.19)	0.544	0.88 (0.72–1.09)	0.238
Muslim	0.88 (0.77–1.09)	0.089	0.86 (0.79–1.54)	0.203	0.85 (0.68–1.06)	0.140	1.03 (0.84–1.25)	0.807
Other	0.90 (0.76–1.08)	0.087	0.31 (0.16–0.61)	0.000	0.98 (0.48–2.02)	0.962	1.06 (0.66–1.68)	0.818
**Family size**								
Less than 4	Ref.		Ref.		Ref.		Ref.	
4–8	0.97 (0.86–1.10)	0.640	0.57 (0.48–0.64)	0.000	0.96 (0.79–1.16)	0.663	0.94 (0.80–1.10)	0.428
More than 8	0.83 (0.69–0.99)	0.033	1.07 (0.83–1.36)	0.851	0.63 (0.46–0.86)	0.004	1.03 (0.82–1.29)	0.830
**Sex of household head**								
Male	Ref.		Ref.		Ref.		Ref.	
Female	0.98 (0.84–1.15)	0.809	1.07 (0.88–1.29)	0.517	0.84 (0.66–1.08)	0.176	1.04 (0.86–1.27)	0.673
**Wealth index**								
Poor	Ref.		Ref.		Ref.		Ref.	
Middle	1.07 (0.92–1.23)	0.382	1.80 (1.46–2.21)	0.000	1.39 (1.12–1.73)	0.003	1.14 (1.01–1.32)	0.006
Rich	1.97 (1.74–2.23)	0.000	2.20 (1.58–3.92)	0.000	2.05 (1.67–2.51)	0.000	1.20 (1.02–1.41)	0.030
**Marital status**								
Single	Ref.		Ref.		Ref.		Ref.	
Married	1.81 (1.27–2.59)	0.001	1.10 (0.70–1.73)	0.690	1.50 (0.89–2.51)	0.126	1.65 (1.11–2.46)	0.014
Widowed	1.21 (0.67–2.20)	0.246	0.60 (0.25–1.43)	0.246	1.27 (0.49–3.27)	0.626	1.22 (0.64–2.32)	0.546
Divorced	1.03 (0.64–1.66)	0.903	1.49 (0.83–2.68)	0.184	1.56 (0.82–2.95)	0.546	0.86 (0.51–1.45)	0.174
**Total children ever born**								
Only one (Ref.)	Ref.		Ref.		Ref.		Ref.	
Two to three	0.73 (0.47–0.87)	0.000	0.53 (0.44–0.63)	0.000	0.52 (0.42–0.65)	0.000	0.81 (0.67–0.99)	0.000
More than three	0.35 (0.26–0.51)	0.000	0.25 (0.21–0.31)	0.000	0.36 (0.27–0.49)	0.000	0.37 (0.22–0.61)	0.000
**Dependency**					**Odds Ratio (OR)**		**P – value**	
Timing of ANC Initiation/ANC contacts					2.030		0.000	

Key: Ref.: Represents reference (baseline) category; COR: Crude Odds Ratio; AOR: Adjusted Odds Ratio.

**Table 6:** Confusion Matrix (Observed vs Predicted).

Observed count	Predicted count				
	After the first trimester and fewer than 4 contacts	After the first trimester and 4 or more contacts	In the first trimester, and with fewer than 4 contacts	At the first trimester and 4 or more contacts	Row total
After the first trimester and fewer than 4 contacts	1400	500	20	33	1953
After the first trimester and 4 or more contacts	460	2150	15	35	2660
In the first trimester, and fewer than 4 contacts	15	20	250	50	335
In the first trimester and 4 or more contacts	10	150	5	745	910
**Column total**	1885	2820	290	863	5492

A model comparison between the null model (without predictors) and the full model (including all covariates) was conducted using bivariate binary logistic regression. The full model worked better because it had lower deviance (11629.15), AIC (11771.15), and BIC (12243.32), and a higher log-likelihood ratio (LLR = −5814.57) than the null model (LLR = −6354.69). These results indicate that including the predictors significantly improves model performance (see Table 4). Therefore, using the full model is important for accurate inference, and the detailed results are presented in Table 5.

### Parameter estimation

In this study, the predictors of maternal education, region, maternal age, wealth index, and total children ever born were significantly associated with both ANC contacts and the timing of ANC initiation. Additionally, place of residence and family size were significantly related to the timing of ANC initiation. Marital status was found to be significantly associated only with ANC contacts. The COR (see Table 5) highlights the unadjusted associations, while the AOR provides the adjusted estimates in the presence of other predictors and the dependence between the two outcomes

The odds of timely ANC initiation and receiving ANC contacts were significantly higher among women aged 25–29 and 30–34 compared to those aged 15–19. Specifically, the adjusted odds ratios (AOR) were 1.715 (95% CI: 1.166–2.520) and 2.250 (95% CI: 1.482–3.417) for timely ANC initiation, and 1.564 (95% CI: 1.148–2.131) and 1.452 (95% CI: 1.037–2.032) for optimal ANC contacts, respectively.

Reproductive-age women residing in regional states of Ethiopia had lower odds of timely ANC initiation compared to those in the capital city, Addis Ababa. For instance, the AORs for timely ANC initiation were 0.343 (95% CI: 0.191–0.619) for Somali and 0.495 (95% CI: 0.329–0.745) for SNNPR. Similarly, women from regional states had lower odds of receiving ANC contacts compared to those in Addis Ababa, with AORs of 0.509 (95% CI: 0.291–0.815) for Amhara, 0.467 (95% CI: 0.280–0.781) for Oromia, 0.390 (95% CI: 0.224–0.680) for Somali, 0.344 (95% CI: 0.204–0.597) for SNNPR, 0.197 (95% CI: 0.074–0.524) for Gambela, and 0.304 (95% CI: 0.096–0.968) for Harari.

Our results indicate that women’s educational status is a significant positive determinant for ANC contacts. Women with primary, secondary, and higher education were 1.376 times (AOR = 1.376; 95% CI: 1.192–1.588), 2.447 times (AOR = 2.447; 95% CI: 1.843–3.248), and 2.940 times (AOR = 2.940; 95% CI: 1.913–4.517) more likely to have ANC contacts compared to uneducated women, respectively. Women with higher education were also 1.602 times (AOR = 1.602; 95% CI: 1.121–2.289) more likely to initiate ANC timely compared to those with no education.

Furthermore, the odds of timely ANC initiation and ANC contacts were higher among women from households with middle and rich wealth status compared to those from poor wealth status. Specifically, the odds were 1.391 times (AOR = 1.391; 95% CI: 1.121–1.726) and 2.047 times (AOR = 2.047; 95% CI: 1.669–2.511) higher for timely ANC initiation, and 1.141 times (AOR = 1.141; 95% CI: 1.007–1.321) and 1.197 times (AOR = 1.197; 95% CI: 1.017–1.409) higher for optimal ANC contacts, respectively.

The likelihood of timing of ANC initiation and ANC contacts among reproductive-age women born 2–3 and with more than 3 children was lower by 0.521 (AOR = 0.521; 95%CI: 0.416–0.651), 0.359 (AOR = 0.359; 95%CI: 0.266–0.485), 0.81 (AOR = 0.810; 95%CI: 0.67–0.99), and 0.371 (AOR = 0.371; 95%CI: 0.22−0) as compared to women having only on child, respectively. The likelihood of timing ANC initiation was lower among reproductive-age women with more than 8 family members (AOR = 0.627; 95%CI: 0.455–0.864) when compared to less than 4 family members. The estimated odds ratio of timing ANC initiation among women who reside in rural areas was lower by 0.790 (AOR = 0.790; 95% CI: 0.652–0.957) as compared to women who reside in urban areas. The odds of ANC contacts among reproductive-age women who are married were 1.651 (AOR = 1.615; 95% CI: 1.109–2.459) times higher than compared of single women, respectively (see Table 5).

### A measure of goodness of fit test

Based on the estimated bivariate binary logistic regression model, the predicted and observed counts for the combination of ANC contacts and timing of ANC initiation were compared. The model achieved a correct classification rate (CCR) of approximately 82.77%, calculated by dividing the total number of correct predictions (1,400 + 2,150 + 250 + 745 = 4,545) by the total number of observations (5,492). This high CCR indicates that the estimated model fits the data well (see Table 6).

## Discussion

This study examined how maternal, household, and community-level characteristics jointly influence two critical components of ANC utilization: ANC contacts (4 or more contacts) and timely ANC initiation (within the first trimester). Using data from the 2019 EMDHS and applying a bivariate binary logistic regression model, we found a significant correlation between these two outcomes. Specifically, women who initiated ANC early were more likely to complete the recommended number of contacts. The model identified maternal age, region, maternal education, wealth index, and number of children ever born as common predictors for both timely ANC initiation and ANC contacts. Furthermore, place of residence and household size were uniquely associated with ANC initiation timing, while marital status emerged as a specific determinant of ANC contacts. The use of a bivariate binary logistic regression approach is particularly valuable as it simultaneously accounts for the interdependence between ANC initiation timing and ANC contacts, providing more efficient and unbiased estimates than separate univariate models.

Our findings indicate that household wealth status is a significant predictor of both ANC contacts and the timing of ANC initiation. Women from wealthier households had notably more ANC contacts and initiated ANC earlier in the first trimester compared to women from poorer households. This finding is consistent with studies from Sub-Saharan Africa [12,39], Ethiopia [11], and Indonesia [40], which found that women from wealthier families were more likely to receive ANC services and start ANC in the first trimester. This association may be attributed to the fact that economic prosperity enhances healthcare access and the ability to cover medical and non-medical expenses related to ANC services during pregnancy [41–44]. Consequently, these findings suggest that wealth status is a crucial determinant of ANC contacts. Lower socioeconomic status often results in limited funds for transportation to healthcare facilities for ANC services. Additionally, women from wealthier families may benefit from better education and greater access to mass media compared to those from poorer families.

Urban women are more likely to initiate ANC on time. This finding aligns with results from other studies [45–47]. Such disparities may be attributed to differences in service accessibility and women’s awareness of ANC services between rural and urban areas [48]. Additionally, reproductive-age women with larger families, those with more than eight members, were less likely to time their ANC initiation properly compared to women with smaller families, those with fewer than four members. This observation is consistent with research conducted in Ethiopia [11,21] and Rwanda [49]. It can be attributed to financial constraints that become more pronounced with increasing family size.

Our study also found significant regional differences in the timing of ANC initiation and the occurrence of ANC contacts across Ethiopia’s administrative regions. Women in regional states are less likely to have ANC contacts and timely ANC initiation compared to women in Addis Ababa. This finding is supported by previous research conducted in Ethiopia [11], as well as in Rwanda [50], Nigeria [51], and Bangladesh [52]. The disparities can be attributed to barriers on both the demand and supply sides, including limited access to services in remote and difficult-to-reach areas, as well as issues related to knowledge, transportation, and affordability. Addis Ababa, as the capital city, benefits from better access to health facilities and greater awareness of maternal health services [11].

In this finding, women with higher levels of education are more likely to have optimal timing for ANC initiation and ANC contacts. This result aligns with previous research [29,39,53,54], which shows that education enhances healthcare utilization and increases knowledge about specific health issues. Education, combined with household wealth and decision-making power, empowers women and consequently boosts their use of maternal healthcare services [55].

In this study, we found that younger women of reproductive age were less likely to initiate ANC contacts on time compared to their older counterparts. This finding is consistent with previous research [56,57]. Younger, unmarried women may be more inclined to delay pregnancy disclosure to avoid potential social repercussions. Additionally, older women are generally more aware of early pregnancy signs and are better at recognizing their pregnancy early [58]. Early pregnancy detection can lead to a more timely initiation of ANC, as suggested by one study [58]. We also observed that pregnant women with more children were less likely to have ANC contacts and timely ANC initiation compared to those with fewer children. This observation aligns with previous research from Ethiopia [11,59], Ghana [60], and Rwanda [61]. Factors such as limited time and resources in larger families, along with increased self-confidence from previous pregnancies and childbirth, may contribute to a reduced use of ANC services [62–64]. Enhancing awareness and improving living conditions for women could lead to increased antenatal care utilization, thereby reducing maternal mortality and morbidity and helping Ethiopia achieve its SDG targets.

### Strengths and limitations of the study

This study has several strengths. First, it used data from the nationally representative 2019 EMDHS, which provides high-quality, population-based information on maternal and child health, household, and community-level factors. The large sample size, based on a multi-stage random sampling method, ensures that the findings are generalizable to the broader population of Ethiopian women of reproductive age. Another major strength is the use of a bivariate binary logistic regression model, which jointly estimates the effects of predictors on two interrelated ANC outcomes: timely initiation and optimal number of contacts. This modeling approach improves estimation efficiency and accounts for potential correlations between the two ANC components, yielding more accurate and informative results than standard univariate models.

However, the study also has limitations. Despite the model’s strength, it relies on cross-sectional data, which restricts the ability to establish causal relationships. Another limitation is the potential for reporting or recall bias inherent in survey-based studies, particularly for self-reported health behaviors and past events like timing and frequency of ANC contacts. Additionally, although efforts were made to include all relevant covariates, there remains a risk of omitted variable bias due to unmeasured confounders. Lastly, although the EMDHS data is high-quality, potential data entry or coding errors in large-scale surveys may introduce some measurement error.

## Conclusion

Despite marked improvements in ANC coverage in Ethiopia in recent years, national targets remain unmet, and significant disparities persist. Women residing in rural areas, those from poorer households, and those with lower educational attainment continue to face substantial barriers to timely and adequate ANC utilization. These inequities highlight the urgent need for targeted advocacy and behavior change communication strategies. Programs should prioritize the most disadvantaged groups, particularly rural, impoverished, and less-educated women, through mass media campaigns, community dialogues, and strengthened health extension services. Furthermore, future studies should investigate how cultural and religious beliefs influence ANC utilization patterns, particularly in regions with diverse religious affiliations. Understanding these sociocultural factors may help tailor more effective, culturally sensitive interventions to improve maternal health outcomes and advance Ethiopia’s progress toward achieving Sustainable Development Goals (SDGs).

## Abbreviations

ANC: Antenatal care
DHS: Demographic health survey
EAs: Enumeration areas
EMDHS: Ethiopian mini demographic and health survey
LIMICs: Low- and middle-income countries
VGAM: Vector Generalized Additive Model
